# Supplementary material for: FERN – a Java framework for stochastic simulation and evaluation of reaction networks
Source: BMC Bioinformatics. 2008 Aug 29;9:356. doi: 10.1186/1471-2105-9-356 (PMC2553347; doi:10.1186/1471-2105-9-356)
Supplement: Additional file 1 — FERN distribution, Version 1.3. This archive contains the FERN source code and binaries as well as documentation and example models in FernML and SBML. [file 1471-2105-9-356-S1.zip › fern/doc/javadoc/fern/simulation/observer/LeapObserver.html]

LeapObserver


---


|  |  |  |
| --- | --- | --- |
| **Overview** \| **Package** \| **Class** \| **Use** \| **Tree** \| **Deprecated** \| **Index** \| **Help** | |  |
| **PREV CLASS**   **NEXT CLASS** | **FRAMES**    **NO FRAMES**     **All Classes** |  |
| SUMMARY: NESTED \| FIELD \| CONSTR \| METHOD | DETAIL: FIELD \| CONSTR \| METHOD |  |


---


## fern.simulation.observer Class LeapObserver

```
java.lang.Object
  fern.simulation.observer.Observer
      fern.simulation.observer.LeapObserver
```

**All Implemented Interfaces:**: GnuPlotObserver

---

``` public class LeapObserver extends Observer implements GnuPlotObserver ```

Observes the amount of given molecule species at each `step`. It particularly
makes sense for the approximative leaping algorithms whose `step`s are
precisely the leaps and you want to know about amount differences after each leap.

This observer does not take repeats into account. If you repeat the simulation,
you will just get the results of the recent run.

**Author:**
:   Florian Erhard

---

**Constructor Summary**

`LeapObserver(Simulator sim,
String... speciesNames)`
  
          Creates the observer with a given simulator and given species


| **Method Summary** | |
| --- | --- |
| `void` | `activateReaction(int mu, double tau, Simulator.FireType fireType, int times)`             Do nothing. |
| `void` | `finished()`             Do nothing. |
| `int` | `getNumLeaps()`             Gets the number of the performed leaps |
| `String[]` | `getStyles()`             Gets the styles for the columns. |
| `void` | `started()`             Clears the recorded data. |
| `void` | `step()`             Record the time and amounts of the species. |
| `void` | `theta(double theta)`             Do nothing. |
| `GnuPlot` | `toGnuplot()`             Creates a new `GnuPlot` object and passes the actual observer data to it. |
| `GnuPlot` | `toGnuplot(GnuPlot gnuplot)`             Passes the actual observer data to a `GnuPlot` object. |
| `String` | `toString()` |

**Methods inherited from class fern.simulation.observer.Observer**

`applyLabelFormat, getLabel, getLabelFormat, getNumSimulations, getPrintWriter, getSimulator, getTheta, getTitlesCommand, print, setLabelFormat, setPrintWriter, setSimulator, setTheta`

**Methods inherited from class java.lang.Object**

`clone, equals, finalize, getClass, hashCode, notify, notifyAll, wait, wait, wait`

**Constructor Detail**

### LeapObserver

```
public LeapObserver(Simulator sim,
                    String... speciesNames)
```

:   Creates the observer with a given simulator and given species

    **Parameters:**: `sim` - simulator: `speciesNames` - names of the species


**Method Detail**

### activateReaction

```
public void activateReaction(int mu,
                             double tau,
                             Simulator.FireType fireType,
                             int times)
```

:   Do nothing.

    :   **Specified by:**: `activateReaction` in class `Observer`
    :   **Parameters:**: `mu` - the reaction which is supposed to fire: `tau` - the time the reaction fires (at this time `Simulator.getTime()` does not necessarily yield the firing time): `fireType` - the type of the firing: `times` - TODO

---


### finished

```
public void finished()
```

:   Do nothing.

    :   **Specified by:**: `finished` in class `Observer`

---


### started

```
public void started()
```

:   Clears the recorded data.

    :   **Specified by:**: `started` in class `Observer`

---


### step

```
public void step()
```

:   Record the time and amounts of the species.

    :   **Specified by:**: `step` in class `Observer`

---


### theta

```
public void theta(double theta)
```

:   Do nothing.

    :   **Specified by:**: `theta` in class `Observer`
    :   **Parameters:**: `theta` - moment in time

---


### toGnuplot

```
public GnuPlot toGnuplot()
                  throws IOException
```

:   **Description copied from interface: `GnuPlotObserver`**
:   Creates a new `GnuPlot` object and passes the actual observer data to it.

    :   **Specified by:**: `toGnuplot` in interface `GnuPlotObserver`
    :   **Returns:**: the created `GnuPlot` object **Throws:**: `IOException` - if gnuplot could not be accessed

---


### toGnuplot

```
public GnuPlot toGnuplot(GnuPlot gnuplot)
                  throws IOException
```

:   **Description copied from interface: `GnuPlotObserver`**
:   Passes the actual observer data to a `GnuPlot` object.

    :   **Specified by:**: `toGnuplot` in interface `GnuPlotObserver`
    :   **Parameters:**: `gnuplot` - the `GnuPlot` object to pass the data to **Returns:**: the `GnuPlot` object **Throws:**: `IOException` - if gnuplot could not be accessed

---


### getNumLeaps

```
public int getNumLeaps()
```

:   Gets the number of the performed leaps

    :   **Returns:**: number of leaps.

---


### getStyles

```
public String[] getStyles()
```

:   **Description copied from interface: `GnuPlotObserver`**
:   Gets the styles for the columns. If you don't want styles, just return null!

    :   **Specified by:**: `getStyles` in interface `GnuPlotObserver`
    :   **Returns:**: styles for the columns

---


### toString

```
public String toString()
```

:   **Overrides:**: `toString` in class `Object`


---


|  |  |  |
| --- | --- | --- |
| **Overview** \| **Package** \| **Class** \| **Use** \| **Tree** \| **Deprecated** \| **Index** \| **Help** | |  |
| **PREV CLASS**   **NEXT CLASS** | **FRAMES**    **NO FRAMES**     **All Classes** |  |
| SUMMARY: NESTED \| FIELD \| CONSTR \| METHOD | DETAIL: FIELD \| CONSTR \| METHOD |  |


---
